# Supporting decision-making by a health promotion programme: experiences of persons ageing in the context of migration

**DOI:** 10.1080/17482631.2017.1337459

**Published:** 2017-06-22

**Authors:** Emmelie Barenfeld, Susanne Gustafsson, Lars Wallin, Synneve Dahlin-Ivanoff

**Affiliations:** ^a^ Department of Health and Rehabilitation, Institute of Neuroscience and Physiology, Sahlgrenska Academy, Centre for Ageing and Health - AgeCap, University of Gothenburg, Göteborg, Sweden; ^b^ Department of Occupational therapy and Physiotherapy, The Sahlgrenska University Hospital, Göteborg, Sweden; ^c^ School of Education, Health, and Social Studies, Dalarna University, Falun, Sweden; ^d^ Department of Neurobiology, Care Sciences and Society, Division of Nursing, Karolinska Institutet, Huddinge, Sweden; ^e^ Department of Health and Care Sciences, The Sahlgrenska Academy, University of Gothenburg, Göteborg, Sweden

**Keywords:** Health literacy, emigrants and immigrants, ageing, person-centred approach, group intervention, health care quality, access and evaluation

## Abstract

This study is part of the Promoting Aging Migrants’ Capabilities programme that applied person-centred group meetings and one individual home visit to prolong independence in daily activities among people ≥70 years who had migrated to Sweden from Finland or the Western Balkan region. With the purpose to understand programme outcomes, the study aimed to explore the participants’ everyday experiences of using health-promoting messages exchanged during the programme. Using a grounded theory approach, 12 persons aged 70–83 years were interviewed six months to one year after their participation in the programme. The participants experienced how using health-promoting messages was a dynamic process of how to make decisions on taking action to satisfy health-related needs of oneself or others immediately or deferring action. Five sub-processes were also identified: *gaining inner strength, meeting challenges in available resources, being attentive to what is worth knowing, approaching health risks, and identifying opportunities to advocate for others*. The results suggest that the programme could develop personal skills to support older people who have migrated to overcome health-related challenges. They further demonstrate the importance of supporting their health literacy before personal resources hinder action, and call for research on programmes to overcome environmental barriers to health.

## Introduction

A growing number of studies highlight the importance of health promotion to support older persons to manage their everyday life and experience health (Beswick et al., 2008; Gustafsson et al., 2012; Huss, Stuck, Rubenstein, Egger, & Clough-Gorr, 2008). Both ageing and migration processes might influence health (Kulla, Ekman, & Sarvimäki, 2010; Marmot et al., 2012; Torres, 2006a); older persons born abroad are therefore a priority population for health promotion programmes. Evaluations of such programmes aimed to increase knowledge and skills of the individual person ageing in a migration context are however sparse (Lood, Häggblom Kronlöf, & Dahlin-Ivanoff, 2015a). Due to the complexity of health promotion programmes, evaluations focusing on the process contributing to programme outcomes are recommended as a complement to outcome evaluation (Craig et al., 2013).

A literature review and meta-analysis (Lood et al., 2015a) indicates that culturally and linguistically adapted health promotion programmes with a person-centred approach support the capability of older persons born abroad to access health promotion. The Promoting Ageing Migrants’ Capabilities (Gustafsson et al., 2015) is one such programme. The programme comprised four weekly group meetings with an inter-professional team followed by an individual home visit. The goal was to increase knowledge about ageing and provide strategies to support older persons born abroad to manage their everyday life and to experience health. Health information was provided in a written booklet and discussed during the meetings. A previous study (Barenfeld, Gustafsson, Wallin, & Dahlin-Ivanoff, 2015) showed that the Promoting Ageing Migrants’ Capabilities programme helped to raise awareness in the targeted population, as health promoting messages were exchanged between both peers and personnel. The exchanged messages supported awareness about how to promote health, and empower human values and abilities (Barenfeld et al., 2015). Thus, health promotion processes were initiated during the programme, by enabling persons to increase control over or improve their health (World Health Organization, 1998). However, for a programme to result in a behavioural change, health promotion processes initiated during a programme also need to be implemented into the person’s everyday life (Baranowski, Fau-Stables, & Stables, 2000).

The capability to implement one´s resources to achieve desired health goals in everyday life may vary from person to person. Older persons born abroad might face both age-related decline in bodily functions (Morley et al., 2013), and migration-related challenges associated with environmental factors necessary to maintain health. Prah Ruger (2010) has identified that a person’s capability to achieve health goals of value is shaped by the interplay between internal and external factors. The internal factors to consider are, for example, health knowledge and health seeking skills. These internal factors can be influenced by Promoting Ageing Migrants’ Capabilities or similar health promotion programmes. The external factors related to one’s environment to consider are social norms, social networks, economic situation, and access to health care services (Prah Ruger, 2010). The external factors identified by Ruger may be influenced by migration-related challenges. Studies have shown that challenges such as loss of social networks and alternation of cultural contexts may influence daily activities and health negatively (Alizadeh-Khoei, Mathews, & Hossain, 2011; Bhugra, 2004). Furthermore, older persons born abroad could be confronted with poorer living conditions compared to native-born persons (Silveira, Skoog, Sundh, Allebeck, & Steen, 2002; Statistics Sweden, 2012). Barriers are also reported related to health care access (Alizadeh-Khoei et al., 2011; Rechel, Mladovsky, Ingleby, Mackenbach, & McKee, 2013). Despite the potential influence from external factors on programme outcomes, there are no studies exploring experienced capabilities to promote health in everyday life among older persons born abroad after their participation in a health promotion programme. In addition, there are no studies exploring the meaning and use of health knowledge and health seeking skills after participation. Therefore, the focus of this paper is to contribute to the understanding of how a health promotion programme is experienced to enable older persons born abroad to maintain health in their everyday context.

Research evaluating health promoting programmes aimed at supporting older persons born abroad to manage everyday life and experience health is sparse (Lood et al., 2015a). More studies are needed to support future programme development and implementation. To understand programme benefits from a participant’s point of view there is a need to investigate how exchanged health-promoting messages promote internal factors over time. In addition, investigations should target how older persons born abroad experience their possibilities to act upon valued health promoting messages in their everyday life. A few recent studies are noteworthy which have explored the experiences of health and how older persons born abroad use their resources to maintain health (Kulla, Sarvimäki, & Fagerström, 2006; Lood, Häggblom-Kronlöf, & Dellenborg, 2015b). However, the studies referred to have not explored the process of integrating health-promoting messages from a programme into everyday life. Thus, experiences of usefulness and possibilities of integrating health-promoting messages from a programme into everyday life, to maintain or improve health, from the perspective of older persons born abroad still merits exploration. Consequently, this study aimed to explore the experiences of using health-promoting messages amongst older persons born abroad 6 months to one year after their participation in the Promoting Ageing Migrants’ Capabilities programme.

## Methods

### Study design and study context

A grounded theory (G.T.) design was used to gain understanding of the process influencing programme outcomes. The present study was guided by the constructivist approach suggested by Charmaz (2006). Central to the constructivist approach is that people including researchers construct their realities, and theoretical renderings are assumed as interpretive portrayals of reality. Thus, in line with Charmaz (2006) the goal was to gain situational knowledge rather than creating general abstract theories. The approach was chosen as it enables exploration of how participants construct their meanings and actions (Charmaz, 2006), as it relates to their everyday life in relation to health-promoting messages.

This study was a part of a larger collaborative project, the Promoting Ageing Migrants’ Capabilities study including independently living people ≥70 years who had migrated to Sweden from Finland or from the Western Balkan region (Gustafsson et al., 2015). Detailed description of the Promoting Ageing Migrants’ Capabilities study, the Swedish welfare system and the district where the study took place is reported elsewhere (Gustafsson et al., 2015). In short, the programme Promoting Ageing Migrants’ Capabilities comprised four weekly group meetings and an individual follow-up home visit. The group meetings aimed to enable the participants to learn from each other, through peer learning (Shiner, 1999) and to exchange health information with an inter-professional-team. The team consisted of a registered nurse, a physiotherapist, an occupational therapist and a qualified social worker. A booklet covering different aspects of self-management of health served as a basis for the group meetings. In line with a person-centred approach (Ekman et al., 2011; Leplege et al., 2007), health promoting actions were implemented by addressing the participants' own life experiences. Both personnel and participants brought their expertise into the group meetings and shared decision-making was applied. Thus, the group meeting was administrated in partnership between the participants and the inter-professional-team. Therefore the content and discussions could vary according to the participants’ experiences, needs and resources. The study took place in a low income suburban district situated in medium-sized city in Sweden, where 50% of the total population was born abroad.

### Sampling and participants

From our previous study (Barenfeld et al., 2015) all 14 participants were invited to purposefully participate in the present study. The following initial sampling criteria were used to reach heterogeneity; age, gender, type of housing, language spoken during the senior meetings and marital status. These participants were aged ≥70 years, performed daily activities independently (Sonn, 1996), lived in ordinary housing in an urban district and had immigrated to Sweden from Finland or the Western Balkan region. Impaired cognition, defined as below 80% of administered items on the Mini Mental State Examination (Folstein, Folstein, & McHugh, 1975), was used as an exclusion criterion. Participation in the Promoting Ageing Migrants’ Capabilities intervention groups (Gustafsson et al., 2015) was an inclusion criterion. Two persons declined to participate. In accordance with G.T. (Charmaz, 2006) saturation was reached when no new properties of the categories emerged during data collection. Interview number eleven and twelve did not contribute to new properties, thus the categories were considered to be saturated. Therefore, twelve participants were included.

Six women and six men, aged 70–83 years, were included. Seven participants had migrated from Finland and five from the Western Balkan region. All had been living in Sweden for at least 11 years, and most for 21 years or more. They had migrated to Sweden for different reasons such as work or education (*n *= 6), family (*n *= 3) or to find safe refuge (*n *= 3). Nearly half of the sample was living alone. Five participants preferred to speak their mother-tongue or experienced difficulties or inability to make themselves understood in Swedish.

### Data collection

In-depth interviews were conducted in participants’ homes between October 2012 and August 2015, to ensure a time variation between participation in the Promoting Ageing Migrants’ Capabilities programme and the interview. The interviews lasted for an average of 57 min (range 22–125 min, median 54 min). The first author conducted seven interviews in Swedish and three research assistants, who were university-educated and fluent in both Swedish and the required language, conducted the remaining five interviews in the participants” native language. An interview guide, based on the findings of our previous study (Barenfeld et al., 2015), was used to facilitate the interviews, along with questions specific to each participant about use of health-promoting messages mentioned during the first interview. To be able to capture the process between participation in the Promoting Ageing Migrants’ Capabilities programme and the interview, the first question was “*Can you please tell me what you think about your participation in the Promoting Ageing Migrants’ Capabilities programme now when (number of months) has passed by?*” The question areas covered: usefulness or non-usefulness, relevance or non-relevance, opportunities or obstacles, remembering or forgetting, changing or not changing, context, individual- and societal-level prerequisites and from thinking to doing or not doing. The interview guide was developed in line with theoretical sampling as the interviews proceeded, resulting in a narrowing of the range of topics to be able to gather specific data (Charmaz, 2006). Probes such as “*Can you please tell me more about that?*” were used. In addition probes formulated as intermediate questions inspired by Charmaz (2006), such as “*Can you tell me what happened next?*” or “*Can you tell me about the events that led to…?*”, were used to capture processes.

The interviews were recorded and transcribed verbatim in Swedish by the first author or in the native language by the research assistants who then translated the interviews into Swedish. As recommended for translation using a constructivist approach, emphasis was placed on grasping the essence of the content rather than word-by-word translation (Croot, Lees, & Grant, 2011; Temple, 2002). Therefore, the research assistants orally explained the essence of the verbatim translations to the first author as well as writing them down.

### Data analysis

Data analysis was guided by Charmaz (2006). Data collection and analysis were conducted in parallel. First, each line was coded close to the data (initial coding). Memos were used to record what was happening in the data and comparisons were made of data within and between interviews. Focused coding was then used to synthesize and explain segments of data with conceptual codes. Conceptual codes were compared and sorted into categories. Memos were used to systematically compare codes and describe how categories emerged (Charmaz, 2006). The analysis was performed in Swedish by the first author in cooperation with the second and fourth authors, who listened to or read the interviews and discussed coding and category development. To minimize translation-related barriers, the research assistants who fulfilled the criteria for translating in a research context (Squires, 2008) were involved as active partners. They verified the essence of the initial and focused coding for the interviews they conducted, wrote memos after each interview, and participated in the discussions during the analysis. This approach to translation is recommended in relation to the method used because both researchers and assistants were considered to contribute to the construction of meaning (Croot et al., 2011; Temple, 2002).

### Establishing trustworthiness

The criteria for evaluating a G.T. study may differ according to the different epistemology within G.T. approaches (O’Connor, Netting, & Thomas, 2008). In the constructivist approach having a reflexive stance is central for ensuring methodological rigor and for improving quality in the findings (Bryant & Charmaz, 2010; Charmaz, 2006). In this study, the authors and research assistants discussed their occupational life experiences and reflected upon how a migration background verses a native background might influence ones interpretation. In addition, findings and interpretations were checked regularly with the participants during the interviews as part of the theoretical sampling recommended (Charmaz, 2006). Finally, deep thick descriptions (Curtin & Fossey, 2007) were used to illustrate that the theory was grounded in data.

### Ethical considerations

The study followed the ethical principles of the Declaration of Helsinki and was approved by the regional ethics review board on 13 December 2012 (registration number T947-12). Informed consent was obtained from the participants. We confirm that all personal identifiers have been removed or disguised so the persons described are not identified through the details of their story.

## Results

### Making decisions to satisfy needs

The core category for the use of health-promoting messages from the Promoting Ageing Migrants’ Capabilities programme in everyday life was the process of *making decisions to satisfy needs*. This process is dynamic and related to experienced health needs of oneself or others. Experiences of health needs fluctuate over time, and are particularly noticed when a discrepancy occurs between what a person wants to achieve and the prevailing individual or contextual conditions. This influences decision-making about taking action now or deferring action, as well as the prerequisites to satisfy health needs when taking action.

**Figure 1.**
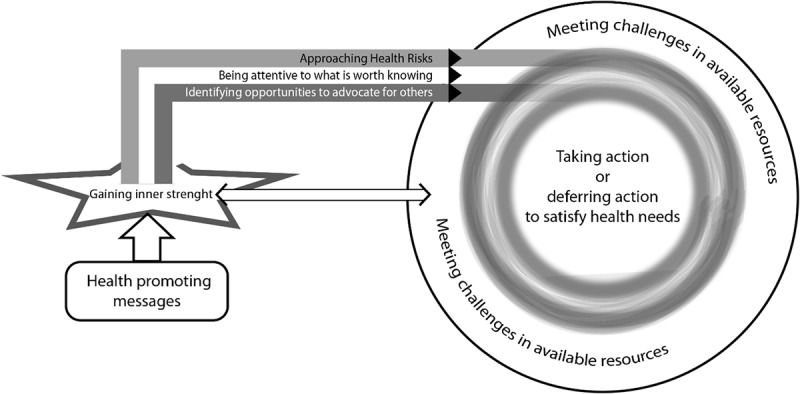
Model visualizing experiences of using health-promoting messages from P.A.M.C. programme to make health decisions in everyday life.

Participants’ experiences of meaning and prerequisites in using health-promoting messages when making and acting upon decisions were described by five interacting sub-processes: *gaining inner strength*, *meeting challenges in available resources*, *being attentive to what is worth knowing*, *approaching health risks* and *identifying opportunities to advocate for others*. These sub-processes are connected to prerequisites identified during the Promoting Ageing Migrants’ Capabilities programme and in everyday life. Health-promoting messages exchanged during the programme lead to experiences of *gaining inner strength*. The inner strength influenced three decision-making processes: *approaching health risks*, *being attentive to what is worth knowing* and *identifying opportunities to advocate for others* (Figure 1). In everyday life, the inner strength worked as either a catalyst or as a soothing mean to take action now or defer action. Decisions were made in relation to three distinct needs connected to each decision-making process: maintaining health, learning more for oneself or others, or advocating for others. Participants’ experiences of using health-promoting messages were shaped by inspiring or obstructing challenges in available resources, in order to satisfy their health needs. Experiences of being able to satisfy needs while taking action in everyday life were interrelated with the opportunity to gain inner strength which accordingly varied over time.

### Gaining inner strength

The sub-process, *gaining inner strength*, reflected the use of the Promoting Ageing Migrants’ Capabilities messages as a power source, where feelings of serenity or a driving force to act could be found. The experience of *gaining inner strength* differed between participants and was shaped by previous experience and the health-promoting messages exchanged during the Promoting Ageing Migrants’ Capabilities programme. These messages contributed to inner strength through knowledge and positive feelings, experienced as being strengthened, inspired and secure. Inner strength was constructed by health-promoting messages regarding safety, community, trusting authorities, the ability to act, human values and the knowledge that you are not alone. Furthermore, participants gained inner strength by experiencing desire to learn, power to keep on fighting, motivation to exercise and courage to ask questions and use the Swedish language. In addition, inner strength was a motivator to seek help or to confide in others about problems. One man told:

*Yes, I got the impression that it’s worth the struggle and that all the know-how is good to have. Especially the bit about not giving up. I don’t really know. You know, I do think more positively [after the programme]. You know, sometimes I’m just lying there with no energy and can’t get out. But you have to fight on anyway [based on the conditions you have]* (Interview 9)

### Meeting challenges in available resources

The sub-process, *meeting challenges in available resources*, was characterized by individual- or environmental-level prerequisites that were facilitating or hindering action (after participation in the Promoting Ageing Migrants’ Capabilities programme). These challenges shaped participants’ experiences of using health-promoting messages to satisfy needs. The use of health-promoting messages was experienced as an inspiring challenge which facilitated actions when individual and environmental resources were available. When participants experienced inadequate physical and psychological resources, the challenges turned to obstacles, which reduced their capability to satisfy health needs. Available resources changed over time due to age related decline or changing prerequisites in the social or physical environment, leading to a variation in the experienced conditions for using health-promotion messages.

Individual resources were experienced as body and mental prerequisites for acting to satisfy needs. Having the bodily condition or possessing the skill to act is a facilitator, inspiring action by positive feedback from the body. In contrast, feeling limited by decreased strength, impaired memory, sadness or inadequate skills to seek and understand information, were experienced as obstacles to taking action to satisfy needs. Environmental-level facilitators were experienced as having access to information and reminders in everyday life, being supported by family and living close to services. Obstructing factors were for example described as waiting times, poor finances and lack of social arenas. One woman described how she encountered barriers in service provision:

*I thought it was plus-minus nothing. I thought it was only a huge fight to get a damned, er, bath board! Why can’t it be like it was, when I had it before, and I come back with it broken. Why can’t I replace it without any fuss. It’s the lack of flexibility in the small things that I think is missing, that’s what I reckon*. (Interview 4)

### Being attentive to what is worth knowing

*Being attentive to what is worth knowing* meant noticing health-promoting messages that were considered important for use now or later. The messages addressed during the Promoting Ageing Migrants’ Capabilities programme were experienced as supporting the process of being attentive to information in everyday life (e.g., health communication in the media and mailings about activities and health services) in a different way than before.

*Yes, you know I didn’t think about it [when I was reading earlier, (sighing)]. They explained to me how important all these things are and all the tips I got there [during the programme]. And then, that there is such information*. (Interview 9)

What was considered worth knowing varied over time in relation to experienced health needs. This influenced decision-making about taking action now or deferring action to learn more for self or others. When taking action, health-promoting messages were used to find out more to be able to approach health risks or advocate for others. Experiences of the ability to act to find out more were shaped by inspiring and obstructing challenges in available resources. One participant described how the written materials received during the Promoting Ageing Migrants’ Capabilities were used as an inspiring challenge by directing the attention to what is worth knowing more about: “*This spurs me into action. I sit down at the computer, go out on the internet, find literature that I think will be useful to me and I search, expanding what I find [in the content of the printed material] even more*” (Interview 5).

Already knowing what is worth knowing supports decisions to defer action: “*I know that I can hold off things, so I think, when I need to. And later on, maybe I start to root around [for more information] if I want to. But not right at the moment*.” Deferring action to find out more indicated that health-promoting messages were not actively used as participants’ were not thinking of them as frequently as before. However, the inner strength gained during the programme challenged the non-use of health-promoting messages. The participants expressed experiences of being reminded of what is worth knowing by events in everyday life or body signals. This facilitated the use of health-promoting messages in decision making. Retaining written material from the Promoting Ageing Migrants’ Capabilities programme was a common strategy participants used to prepare themselves to find out more later on.

### Approaching health risks

The sub-process, *approaching health risks*, was characterized by being able to understand and recognize health risks in new ways after the Promoting Ageing Migrants’ Capabilities programme. This influenced *making decisions to satisfy needs*. Health risks are described as factors or situations leading to a threat to health by jeopardizing the ability to manage everyday life, keeping the mind and body in shape, and staying healthy and injury-free. Health risks differ between participants and fluctuate over time, therefore affecting decisions to act. Participants used health-promoting messages to approach health risks in three different ways: starting to act in new ways, striving for health as before, or waiting for needs to satisfy before acting. The first two ways of approaching health risks differed depending upon whether it was related to a new or old habit. However health-promoting messages were used in a similar way by *approaching health risks* through being physically or socially active, taking action to improve safety, and implementing habits to stay healthy (e.g. simplifying daily activities or changing eating habits).

The possibilities participants’ experienced to approach health risks were shaped by *meeting challenges in available resources*. One man told of how he approached health risks using health-promoting messages to adjust his level of exercise to available inner resources:

*You know, previously I was able to walk for at least half an hour and so-on [which I can’t], but now I read that it’s also important that I walk around the house and at home. My lawn is very soft and there’s a lower risk of getting foot ulcers. And then if I do fall over on the grass, it’s not a big deal*. (Interview 9)

When no need to approach health risks was identified, action was deferred. One participant who had not identified any health risks at the time described the meaning of health-promoting messages in his decision-making about fall prevention:

*We [husband and wife] haven’t done anything actively after this… But we’re encouraged to have an opinion about a lot of things and, and… It hasn´t been lost in any way, no. I think the time [to use it] may yet come*. (Interview 6)

### Identifying opportunities to advocate for others

*Identifying opportunities to advocate for others* was another way participants used health-promoting messages. This meant identifying needs among others and identifying opportunities to help them satisfy these needs. Advocating for others was experienced as a strategy to achieve better health for oneself and for others (e.g., family, friends, neighbours or relatives). It was common for older persons born abroad to experience an inner drive to advocate for others even before the Promoting Ageing Migrants’ Capabilities, by contributing to the community or helping others. Health-promoting messages were used to identify opportunities to act to satisfy previously known needs as well as to identify new opportunities to advocate for others. New opportunities were identified by recognizing others’ need for social contact and noticing that others had knowledge gaps.

Identified needs influenced *making decisions to satisfy needs*. One way to advocate for others was taking action to enable other older persons to share health-promoting messages through participation in the Promoting Ageing Migrants’ Capabilities programme or similar programmes. Participants experienced this as promoting participation in the Promoting Ageing Migrants’ Capabilities programme, planning for study circles in local clubs and groups, or helping staff to recruit participants to the Promoting Ageing Migrants’ Capabilities programme. Advocating for others was also characterized by actions related to disseminating health-promoting messages (i.e. by sharing written material, informing others of health risks, or guiding others to satisfy their needs). One participant used health-promoting messages to identify opportunities to guide a friend to health services:

*There must be something [to do], I thought when I saw him walking. You have to remember that the foot is bad, but your thighs, you’re losing your thigh muscles. What would happen if you couldn’t get to that physiotherapist? I think he was at the physiotherapist’s a few times. It was where visits to the training place started, whichever order that was in. But he goes there [the training place] and he’s been going there all winter, he still goes and he thinks it’s good*. (Interview 4)

## Discussion

This study provides valuable new insights as to how older persons born abroad experienced the meaning and opportunities of using messages from a health promotion programme in order to achieve valuable health goals in their everyday life. The main finding was how the health-promoting processes that started during the Promoting Ageing Migrants’ Capabilities programme were used as tools in decision-making to satisfy health needs for both self and for other persons. Additionally, the study found that the ability to act upon valuable decisions could be facilitated or hindered due to available resources related to the ageing process or environmental prerequisites.

An important contribution from our study is that the Promoting Ageing Migrants’ Capabilities programme supported decision-making about taking action now or deferring action to satisfy health needs. Both age and migration may contribute to frailty (Brothers, Theou, & Rockwood, 2014; Morley et al., 2013), a diminished ability to respond to stress resulting in a vulnerability to poor health outcomes. Missed opportunities to benefit from positive contributions to health in the country of residence might explain higher levels of frailty among older persons who have migrated (Brothers et al., 2014). The importance of reaching out with health promotion in an early stage, before a person becomes frail, is known to be important to support older persons to manage everyday life (Stuck, Minder, & Peter-Wuest, 2000). From a person-centred point of view, this doesn´t automatically mean that health promotion programmes should result in behavioural change. Rather programmes should provide prerequisites for persons to prioritize and make health decisions (Fors, 2014). According to the participants, the inner strength gained during the Promoting Ageing Migrants’ Capabilities programme supported decision-making as they experienced empowerment in *being attentive to what is worth knowing*, *approaching health risks* and *identifying opportunities to advocate for others*. These findings demonstrated that the Promoting Ageing Migrants’ Capabilities programme supported access to, and understanding of, health information during the programme and in everyday life.

Previous studies (Behm, Ivanoff, et al., 2013; Behm, Zidén, et al., 2013) have found that there may be psychological barriers to assimilating information early when that information is not perceived to apply to oneself, or is difficult to accept as it may not apply at the present time. Our results support this argument, as participants’ experiences of their own needs or those of others were important for decisions about taking action now or deferring action. It is previously shown that factors other than knowledge influence why people do or do not make behavioural changes to promote health (Gordon, 2002). On the basis of this knowledge health promotion messages should be designed to influence the perceptions of both risks and self-efficacy (Gordon, 2002), which is demonstrated in our categories.

In addition, health-promoting messages should show the benefits of following the messages and encourage persons to overcome obstacles in the social and physical environment (Gordon, 2002). In our study, valuable health goals experienced by older persons born abroad are expressed in relation to three distinct needs: to maintain own health, to learn more for oneself or others, and to advocate for others. These goals are in line with previous research targeting both older persons with and without migration experiences. Previous research has demonstrated the value for both native born Swedes and older persons born abroad of being able to manage everyday life (Fange & Ivanoff, 2009; Kulla et al., 2010; Lood et al., 2015b). In addition, the goal of being able to support other persons’ well-being to experience health is in line with another study exploring experiences of health among older persons born abroad (Lood et al., 2015b). In retrospect, our study adds knowledge about how a health promotion programme could support persons to achieve these valuable health goals. These findings may help to guide the development, implementation and evaluation of health promotion programmes available to older persons born abroad. In addition, tackling the lack of availability of health promotion support requires attention, as it may lead to inequalities.

One way of interpreting our results is that the Promoting Ageing Migrants’ Capabilities programme influenced participants’ health literacy. Health literacy entails knowledge, motivation, and competence to access, understand, appraise and apply health information to make decisions (e.g., health promotion to stay healthy) and is developed through the interaction between the environment and the person (Sørensen et al., 2012). The category *being attentive to what is worth knowing* found in our study demonstrated that the programme supported decision-making by directing the person’s attention towards information of importance to be able to approach health risks or advocate for others. It is already recognized that health literacy is linked to health outcomes (Berkman, Sheridan, Donahue, Halpern, & Crotty, 2011), and that health-promoting programmes may be used to support health literacy (Levasseur & Carrier, 2012). However, to the best of our knowledge, our study is the first to show how a health promotion programme is experienced to support health literacy in everyday life among older persons ageing in the context of migration. The experienced need to learn more for oneself or others also calls for future studies which explore and evaluate if and how health information retrieval skills should be integrated as a component in health promotion programmes.

Another important finding is the experienced possibilities to use health-promoting messages to satisfy health needs. The category *meeting challenges in available resources* showed that contextual resources such as access to health care services and social networks as well as personal prerequisites shaped the possibilities for action. Although our results showed that the Promoting Ageing Migrants’ Capabilities programme might bridge barriers to *making decisions to satisfy health needs*, participants also experienced a lack of effective opportunities to achieve and act towards desired goals. Thus, even if the persons knew what to do or how they wanted to act, they were sometimes limited by bodily and mental conditions such as decreased strength, impaired memory and inadequate skills to seek and understand further information. In these situations, environmental factors such as availability to social network or access to health care services could work as either a facilitator or barrier. This supports the view that health literacy is a complex phenomenon dependent on multidimensional interrelations in the social or cultural context (Mårtensson & Hensing, 2012). Similar to previous studies, our results demonstrate the importance of age-friendly environments (Beard et al., 2015), more accessible health care services (Alizadeh-Khoei et al., 2011; Rechel et al., 2013), and to empower older persons born abroad to use their innate capabilities to promote health (Lood et al., 2015b). Thus, both personal and environmental resources should be targeted to enable older persons born abroad to use health-promoting messages to act upon valuable health goals.

### Methodological limitations

Older persons born abroad are often excluded from research because of language barriers (Hussain-Gambles, Atkin, & Leese, 2004), which is a reason for the lack of knowledge regarding this population. The literature describes the need to use various languages during analysis as a limiting factor (Squires, 2008). However, for our study, it was necessary to include people with different language skills and migration experiences. This also resulted in the heterogeneity of initial sampling criteria, which is recommended (Hallberg, 2006). To address the methodological challenges of translating data, we involved research assistants as active partners, able to validate data during data collection and analysis. This approach improved the quality of the findings by enabling a reflexive stance, for both researchers and translators (Charmaz, 2006; Temple, 2002).

The interviews were performed 6 months to 1 year after participation in the programme. This might be a methodological limitation due to potential recall problems as the older persons were asked to share experiences representing a time span on up to one year. However, performing the interviews in retrospect also enabled the study of the use of health-promoting messages over time, which is unique to this study. The fact that persons from our previous study (Barenfeld et al., 2015), conducted shortly after the participation, were re-interviewed facilitated the interview process and experiences from the previous interviews were used as a memory support. There was a wide range of interview time. The use of theoretical sampling is one explanation for this. As interviews proceeded, the range of topics to gather specific data was narrowed to support development of theoretical frameworks (Charmaz, 2006).

According to Charmaz (2006), an analysis is contextually situated in time, place, culture, and situation. Therefore, the present findings must be understood with regard to the context and specific sample of the study. When interpreting results, it is important to be aware that older persons born abroad are a heterogeneous population (Torres, 2006b). Although, the results might be applicable to older persons who have migrated to countries other than Sweden or older persons without migration experiences, the local context may have influenced participants’ experiences of using health-promoting messages. In the Nordic countries, older persons born abroad have the same legal rights as native born persons (Blackman, 2000; Graham, 2002), but there might be differences in living conditions such as socio economics (Statistics Sweden, 2012). Previous studies show that access to activity and participation might be hindered among persons who have migrated (Bennett, Scornaiencki, Brzozowski, Denis, & Magalhaes, 2012; Santos-Tavares & Thorén-Jönsson, 2013) and that there might be barriers for access to health-information (Kreps & Sparks, 2008). Often the hindrances concern linguistically or culturally aspects but limited availability to social networks might also impact (Santos-Tavares & Thorén-Jönsson, 2013). Therefore, the inclusion of participants strived to reach heterogeneity due to age, gender, living conditions, civil status and language skills. To enhance the transferability of our findings, we provided descriptions of participants’ characteristics and referred to characteristics of the study context.

## Conclusion and implications

Our results contribute to current knowledge by showing how a health promotion programme supported health literacy in everyday life among older persons born abroad. Health-promoting messages from the Promoting Ageing Migrants’ Capabilities programme were used in making decisions to act now or defer action for later to satisfy needs in relation to one’s own or other persons’ health. This suggests that the Promoting Ageing Migrants’ Capabilities programme was successful in developing personal skills to support health choices among older persons ageing in the context of migration. The programme offered tools to satisfy health needs by directing attention to information of importance to be able to approach personal health risks or advocate for others’ health. However, the ability to act to satisfy health needs was shown to be both facilitated and hindered by experienced availability of personal and environmental resources. Therefore, both personal and environmental resources should be targeted to enable older persons born abroad to use health-promoting messages to satisfy health needs in everyday life.

### Implications for policy and practice

- Increase the skills of older persons born abroad to take advantage rights and opportunities within health services.
- Programmes should promote using personal resources to bridge environmental barriers in relation to (1) health maintenance, (2) advocate for others health and (3) for further information retrieval.
- To support older persons born abroad to use health-promoting messages in everyday life health promotion programmes should identify each person’s internal and external resources to act upon valuable health goals.
